# Supplementary material for: Splice-Junction-Based Mapping of Alternative Isoforms in the Human Proteome
Source: Cell Rep. Author manuscript; Available in PMC 2020 Jan 15. (PMC6961840; doi:10.1016/j.celrep.2019.11.026)
Supplement: 2 [file NIHMS1546469-supplement-2.zip › DF1/PXD010154/Heart/Heart_33_UQCRH_MLTESGDPEEDPLTTVR.pdf]

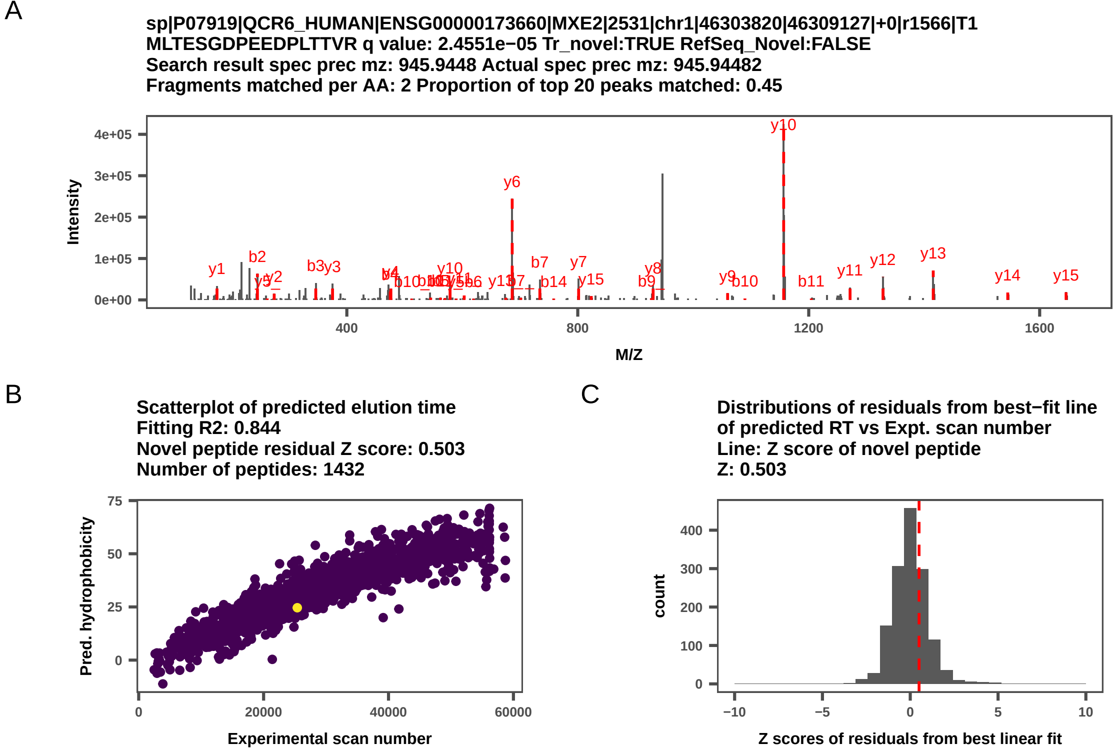
A
sp|P07919|QCR6_HUMAN|ENSG00000173660|MXE2|2531|chr1|46303820|46309127|+0|r1566|T1
MLTESGDPEEDPLTTVR q value: 2.4551e−05 Tr_novel:TRUE RefSeq_Novel:FALSE
Search result spec prec mz: 945.9448 Actual spec prec mz: 945.94482
Fragments matched per AA: 2 Proportion of top 20 peaks matched: 0.45
Intensity
4e+05
3e+05
2e+05
1e+05
0e+00
y10
y6
y1
b2
y2
b3
y3
b7
y7
y15
y8
b9
y9
b10
b11
y11
y12
y13
y14
y15
b14
400
800
1200
1600
M/Z
B
Scatterplot of predicted elution time
Fitting R2: 0.844
Novel peptide residual Z score: 0.503
Number of peptides: 1432
Pred. hydrophobicity
75
50
25
0
0
20000
40000
60000
Experimental scan number
C
Distributions of residuals from best−fit line
of predicted RT vs Expt. scan number
Line: Z score of novel peptide
Z: 0.503
count
400
300
200
100
0
−10
−5
0
5
10
Z scores of residuals from best linear fit
